# A Unique Presentation of an Infected Renal Cyst: A Case Report and Literature Review

**DOI:** 10.7759/cureus.47966

**Published:** 2023-10-30

**Authors:** Perla Mansour, Lama Ammar, Eric O. Gomez Urena, Andrew Chow, Mohamad El Labban

**Affiliations:** 1 School of Medicine, American University of Beirut, Beirut, LBN; 2 Infectious Diseases, Mayo Clinic Health System, Mankato, USA; 3 Radiology and Imaging, Vascular Medicine, Mayo Clinic Health System, Mankato, USA; 4 Internal Medicine, Mayo Clinic Health System, Mankato, USA

**Keywords:** computed tomography-guided aspiration, sepsis, cyst aspiration, interventional radiology, infected renal cyst, renal cyst

## Abstract

Renal cysts are prevalent conditions and are often diagnosed incidentally. The infection of renal cysts is an uncommon presentation. It is even more rare in solitary simple cysts than in autosomal dominant polycystic kidney disease (ADPKD). Patients with infected renal cysts can have variable presenting symptoms; however, almost universally, they have flank pain. Here, we report a case of a solitary renal cyst infection in the absence of flank pain, a relatively rare condition. A 60-year-old male patient presented to our emergency department (ED) for ongoing periumbilical/lower abdominal pain, chills, and high-grade fever. He was initially seen in urgent care and thought to have a urinary tract infection (UTI). He was discharged on trimethoprim-sulfamethoxazole (TMP-SMX). He was hemodynamically stable in the ED and did not have flank pain. Urine culture showed *Escherichia coli*. Computed tomography (CT) showed changes concerning for possible early pyelonephritis of the right kidney area and an enlarged right upper pole renal cyst compared to previous imaging. The urology team was consulted, and the enlarging cyst was considered secondary to hemorrhage. The patient continued to have high-grade fevers and worsening abdominal pain during his stay despite being on culture-directed intravenous antibiotics. Consequently, the cyst was aspirated, and cultures grew *E. coli* with a similar antimicrobial susceptibility pattern as the one found in the urine. After the procedure, the fever and abdominal pain significantly improved. This case report describes a patient with an infected solitary renal cyst with a unique presentation. Imaging modalities can be misleading and delay the diagnosis. Appropriate source control via cyst aspiration and/or drain insertion is crucial for successful treatment.

## Introduction

Renal cysts, a common occurrence especially among the geriatric population, can be found in approximately 50% of individuals aged 50 and above [1]. The etiology of these cysts remains elusive, yet they are widely considered acquired lesions, possibly originating from diverticula located within the convoluted tubules and collecting ducts of the renal system [2]. In 1986, Bosniak introduced a comprehensive classification system consisting of five categories (I, II, IIF, III, and IV) predicated upon the morphological attributes and enhancement patterns exhibited by renal cysts [3,4]. This classification schema serves the purpose of estimating the risk of malignant transformation [4]. Generally, uncomplicated, simple renal cysts necessitate no intervention unless they cause symptoms or complications [5]. Only a scant 8% of renal cysts become symptomatic, demonstrating clinical presentations from flank pain, palpable flank masses, hypertensive episodes, hematuria, intracystic hemorrhage, infectious episodes accompanied by fever, pelvicalyceal obstruction culminating in hydronephrosis, gastrointestinal disturbances, and, rarely, cyst rupture [5]. This report presents a unique presentation of an infected simple renal cyst. The authors also conducted a scholarly search for infection of renal cysts on Pubmed and Ovid MEDLINE. 

## Case presentation

A 60-year-old male patient presented to the emergency department (ED) for worsening nausea and abdominal pain. Past medical history was significant for tobacco dependence, coronary artery disease, gastroesophageal reflux disease (GERD), sialadenitis, stable renal cyst, and obesity. The patient also had a history of a chronic urethral stricture that required intermittent self-catheterization, most recently three weeks before presentation. He denied any previous history of a urinary tract infection (UTI). One week before the presentation, he developed pain in the lower abdomen at the end of urination, dark urine with “chunks,” and chills. The evaluation revealed a normal set of vital signs without a fever, a physical exam with lack of abdominal and costovertebral (CVA) tenderness, a urine analysis with pyuria and hematuria, and neutrophilic leukocytosis. Suspicion at that time was that the patient had a UTI. He was started on a planned 14-day course of trimethoprim-sulfamethoxazole (TMP-SMX). No urine culture was collected.

**Figure 1:**
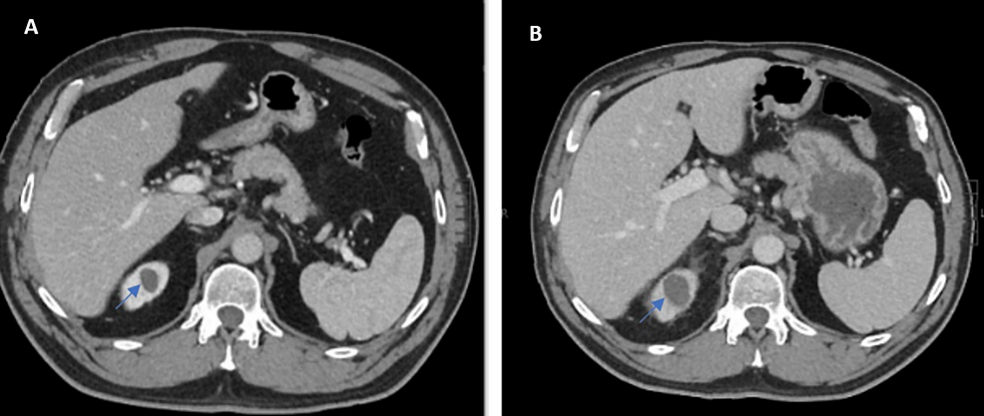
(A) CT scan showing an 18 mm right renal cyst (three years before presentation); (B) CT scan showing a 26 mm right renal cyst in addition to increased density and perinephric fat stranding (at the time of presentation).

After starting TMP-SMX, he noted initial improvement; however, after a few days, he developed persistent chills with rigors but did not measure his temperature. The patient eventually presented to the ED for acute onset of worsening abdominal pain, nausea, and bilateral lower back pain. He denied flank pain and urinary symptoms, such as dysuria and frequency. On presentation, he had sinus tachycardia (heart rate 122) but was otherwise afebrile (37°C) and hemodynamically stable. The physical exam again showed periumbilical abdominal tenderness and negative CVA tenderness bilaterally. The initial workup displayed a normal white blood cell (WBC) count and elevated creatinine level. C-reactive protein (CRP) was significantly elevated at 212.4 mg/L. The urine analysis showed persistent pyuria, resolved hematuria, and elevated specific gravity with hyaline casts. The patient had a computerized tomography (CT) scan of the abdomen and pelvis, which showed changes concerning possible early pyelonephritis in the right kidney and right upper pole renal cyst measuring 26 mm in greatest diameter, which had increased in size (18 mm) from three years earlier. The density of the cyst had also increased from 12.8 Hounsfield units (HU) to 22 HU (uncomplicated fluid 0-20 HU) (Figure 1).

There was some speculation as to whether or not the patient may also have a possible bowel obstruction; therefore, he had a nasogastric tube placed. Upon further evaluation, general surgery ruled out intestinal obstruction, and the nasogastric tube was removed. Ceftriaxone was started and the patient was admitted to the hospital for further evaluation and management.

**Figure 2:**
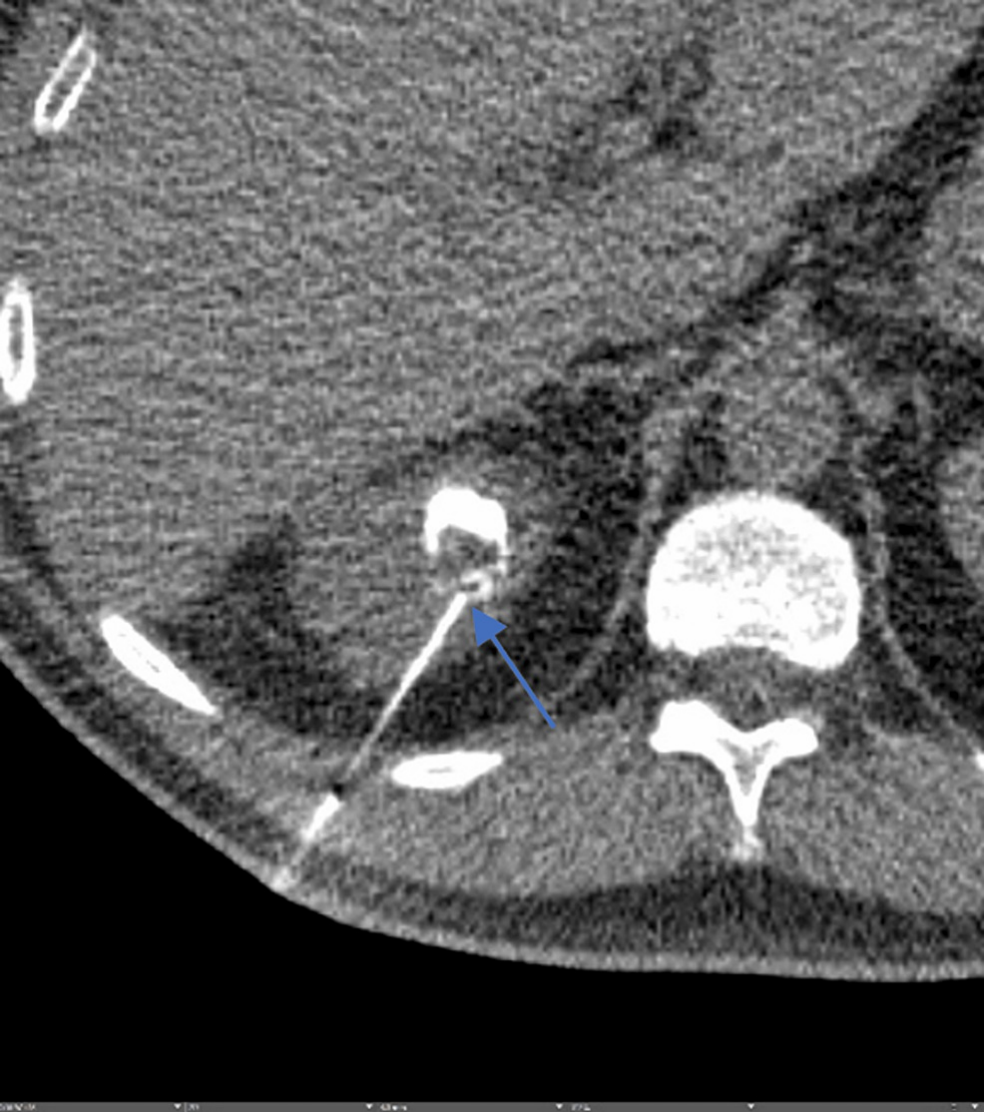
CT-guided aspiration. Arrow: Needle inside the cyst with contrast injected into the cyst cavity and small amount of debris/hemorrhage within the cyst

During his stay, the patient continued to experience high-grade fevers (>39°C) despite broadening antibiotics to piperacillin-tazobactam. Blood cultures remained negative. Urine culture grew *Escherichia coli* resistant to TMP-SMX and ampicillin but sensitive to piperacillin/tazobactam and fluoroquinolones. As per the CT abdomen’s report, the enlarging cyst was considered secondary to hemorrhage. For further evaluation, the urology team was consulted, who confirmed that the cyst was unlikely to be infected and the increase in size was likely secondary to hemorrhage. Decision was made to defer aspiration of the cyst at that time. In the following 36 hours, the patient continued to have high-grade fevers and generalized abdominal pain; consequently, the decision was made to aspirate the cyst. The interventional radiologist aspirated 2 mL of pus from the cyst (Figure 2).

Given the small size of the cyst, a drain was not able to be placed, and instead, the cyst cavity was irrigated multiple times with saline. The renal cyst fluid culture grew *E. coli* with the same antimicrobial susceptibility pattern as the *E. coli* isolate found in the urine. Following the procedure, the fever resolved, along with significant improvement in abdominal pain and nausea. The patient was discharged on oral antibiotics on the second day post-procedure. The infectious disease team recommended levofloxacin 500 mg daily for a total of 14 days from the date of the aspiration. At a follow-up appointment with his primary care provider nine days post-discharge, the patient did not complain of abdominal pain, nausea, or fever. CRP had remarkably improved (12.2 mg/L).

## Discussion

Renal cysts can occur as a solitary entity or in the context of autosomal dominant polycystic kidney disease (ADPKD). ADPKD is one of the most prevalent hereditary disorders globally and represents the most common form of renal cystic disease. Symptoms of ADPKD include early-onset hypertension, hematuria, palpable flank masses, renal insufficiency, and progressive decline in renal function [4]. One of the well-known complications of ADPKD is a kidney cyst infection, with incidence estimated at around one per 100 person-years. Renal cyst infection is a serious complication of ADPKD that is often difficult to treat and may lead to mortality [6]. Renal cyst infection should be considered in an ADPKD patient with acute abdominal pain and fever [6]. Cyst infection may result from ascending UTIs, bloodstream infections, or direct exposure to the simple renal cyst during biopsy and operation [6,7]. Pyuria is frequently absent, which might favor hematogenous spread as a mechanism of infection [6].

In contrast to infection in the setting of ADPKD, the infection of a simple renal cyst is a rare occurrence. In most cases, diagnosing an infected renal cyst by imaging and clinical presentation can be challenging. The gold standard for diagnosing renal cyst infection remains cyst aspiration showing the presence of bacteria and an abnormal WBC count suggestive of infection. However, obtaining a cyst aspirate is not always possible secondary to the small size or the location of the cyst precluding percutaneous access [8]. A study by Latinga et al. that evaluated reports between 1948 and 2014 found that a better diagnostic approach would be combining clinical features, such as fever and flank pain, with biochemical parameters, like CRP and WBC count [8]. While uninflamed kidney cysts are primarily asymptomatic, infected ones almost always present with acute flank pain. While our patient presented with elevated CRP and WBC count, there was a lack of flank pain. Imaging can be of equivocal value in definitively diagnosing infected renal cysts as hemorrhage cysts, or proteinaceous cysts can also raise the density of the renal cyst [9]. The diagnostic interpretation of a CT is also highly radiologist dependent. This was a factor contributing to the delay in the diagnosis in our patient and why his case needs to be highlighted. Each imaging modality has different specificity and sensitivity; while using a Gallium scan has a specificity of just about 50%, MRI can raise the specificity to 84.9% [9,10].

Gram-negative bacilli are the most common organism associated with infected renal cyst, with *E. coli* being present in 75% of cases [11]. This is consistent with the results of our patient’s urine and cyst fluid cultures. A study was conducted to investigate the causative organisms responsible for cyst infections in ADPKD patients between 2004 and 2014 [12]. *E. coli* was the most dominating organism in cystic fluid cultures, followed by *Klebsiella* spp. Urine cultures are unreliable as kidney cysts are usually disconnected from the nephrons and would most likely be negative in the presence of an infection [13].

Upon reviewing some cases reported in the literature, the most common unique characteristic shared between the cases was the presence of an unusual organism. Some of them are *Proteus mirabilis,* which induced an emphysematous infection [14], *Campylobacter jejuni* [15], and *Desulfovibrio* [16]. One study showed a hematogenous route of transmission [17]. Another reported a very rare and serious complication, the development of a brain abscess [18]. While each of these case reports displayed something interesting and unique, all of these patients experienced a certain degree of flank pain as opposed to our patient, making him unique. Similar to what we observed in our patient, blood cultures are usually negative in the setting of infected renal cysts [16,19]. In a case series of 14 patients with cystic infections in ADPKD, only four out of the 14 patients had positive blood cultures [20].

Treatment strategies include antimicrobials, percutaneous aspiration/drainage, and resection via surgery. They can be used individually or in combination. A systematic review was conducted to compare these three methods in ADPKD patients. In almost 80% of cases, antimicrobials were the initial treatment option; however, they were associated with a higher rate of failure than the other strategies. The percutaneous method has been significantly used more frequently in recent years, often combined with antimicrobials. It consists of puncture, aspiration, and possible drain insertion into the cyst. Of all the cases initially treated via a percutaneous approach, none had a recurrence of infection. In fact, only 7% of all cyst infections had returned, the majority being initially treated with antimicrobials [11]. Some indications include fever persisting for more than one to two weeks despite appropriate antibiotics therapy, exceeding 5 cm in diameter [19], severe cyst infections that might lead to sepsis, disseminated intravascular coagulation, or infection with repeated episodes. Adverse events are rare. In a study that included 1,038 cases of cyst drainage, there were only 29 documented events overall (2.7%) with possible complications including pleuroperitoneal fistula, bile fistula, peritonitis, and development of a pneumothorax [10].

## Conclusions

The infection of renal cysts is not very common as most cysts are asymptomatic and rarely cause any complications. When renal cyst infection occurs, early diagnosis and appropriate management can expedite recovery in patients. The infection of solitary cysts is rare compared to those seen in ADPKD. Medical management includes antibiotic therapy. Cyst drainage via percutaneous CT drainage is an emerging treatment modality when patients do not improve with medical therapy. This report highlights infected renal cysts' different clinical presentations and diagnostic challenges.
